# An RNA Aptamer Provides a Novel Approach for the Induction of Apoptosis by Targeting the HPV16 E7 Oncoprotein

**DOI:** 10.1371/journal.pone.0064781

**Published:** 2013-05-30

**Authors:** Clare Nicol, Özlem Cesur, Sophie Forrest, Tamara A. Belyaeva, David H. J. Bunka, G. Eric Blair, Nicola J. Stonehouse

**Affiliations:** School of Molecular and Cellular Biology, Faculty of Biological Sciences and Astbury Centre for Structural Molecular Biology, University of Leeds, Leeds, United Kingdom; Albert Einstein College of Medicine, United States of America

## Abstract

**Background:**

Human papillomavirus 16 (HPV16) is a high-risk DNA tumour virus, which is a major causative agent of cervical cancer. Cellular transformation is associated with deregulated expression of the E6 and E7 oncogenes. E7 has been shown to bind a number of cellular proteins, including the cell cycle control protein pRb. In this study, RNA aptamers (small, single-stranded oligonucleotides selected for high-affinity binding) to HPV16 E7 were employed as molecular tools to further investigate these protein-protein interactions.

**Methodology/Principal Findings:**

This study is focused on one aptamer (termed A2). Transfection of this molecule into HPV16-transformed cells resulted in inhibition of cell proliferation (shown using real-time cell electronic sensing and MTT assays) due to the induction of apoptosis (as demonstrated by Annexin V/propidium iodide staining). GST-pull down and bead binding assays were used to demonstrate that the binding of A2 required N-terminal residues of E7 known to be involved in interaction with the cell cycle control protein, pRb. Using a similar approach, A2 was shown to disrupt the interaction between E7 and pRb *in vitro*. Furthermore, transfection of HPV16-transformed cells with A2 appeared to result in the loss of E7 and rise in pRb levels, as observed by immunoblotting.

**Conclusions/Significance:**

This paper includes the first characterisation of the effects of an E7 RNA aptamer in a cell line derived from a cervical carcinoma. Transfection of cells with A2 was correlated with the loss of E7 and the induction of apoptosis. Aptamers specific for a number of cellular and viral proteins have been documented previously; one aptamer (Macugen) is approved for clinical use and several others are in clinical trials. In addition to its role as a molecular tool, A2 could have further applications in the future.

## Introduction

Human papillomaviruses (HPVs) are DNA tumour viruses that infect keratinocytes in epithelia. More than 100 types have been identified [1], [2] and are classified as either low-risk or high-risk, depending on the associated risk of cancer development. HPV16 is a high-risk virus responsible for approximately 50% of cervical cancers [3], [4]. The virus infects cells in the basal layer of cervical epithelia at sites of wounding. Although the majority of infections are cleared by the immune system, persistent infection can lead to development of cancer. Cellular transformation is initiated through deregulated expression of the viral oncogenes E6 and E7 [5], [6]. High-risk E6 has been shown to promote degradation of the tumour suppressor p53 [7], [8], while E7 binds and destabilises the cell cycle control protein pRb [9], [10]. E7 binds the hypophosphorylated form of pRb, inhibiting its interaction with E2F transcription factors, leading to deregulated progression to the S-phase of the cell cycle [11]. In addition to this well characterised role, E7 has been shown to interact with more than 30 other cellular proteins including p300 and TATA binding protein (TBP) [12], [13]. We have recently reported the selection of RNA aptamers to HPV16 E7 for use as novel molecular tools [14]. Aptamers are short nucleic acids that fold into complex three dimensional structures and bind target molecules in a conformation-dependent manner. Aptamers have potential to rival antibodies in many applications due to their high binding affinity and ability to discriminate between closely related targets. Furthermore, they can be very small (therefore reaching targets that may be inaccessible to antibodies). Nucleic acid aptamers are single-stranded oligonucleotides whereas peptide aptamers are composed of a variable peptide loop attached to a protein scaffold. The former have the advantage in that they are able to undergo multiple rounds of denaturation and re-folding. In addition, they are non-immunogenic and can be synthesised to very high purity and modified in a number of ways to increase their stability, for example by inclusion of modified nucleotides [15], [16], [17]. They can be produced *in vitro* to virtually any target by a process known as Systematic Evolution of Ligands by Exponential Enrichment (SELEX) [18], [19], [20] and delivered to live cells by lipofection. In some cases, certain aptamers have also been shown to be internalised by receptor-mediated endocytosis. This opens up many avenues for development of novel therapeutics as well as tools for the investigation of protein function [21], [22]. Unlike siRNAs which act at the level of mRNA to affect protein expression, nucleic acid aptamers act directly on protein molecules, thus allowing finer control. Such aptamers have been generated to a variety of viral proteins including the HCV protease NS3 [23] and polymerase NS5B [24], SARS coronavirus helicase [25], H5N1 influenza HA [26], FMDV 3D polymerase [27] and several HIV proteins including gp120, reverse transcriptase and the trans-activator protein Tat [21].

Our E7 RNA aptamers [14] were stabilised against nucleases and spontaneous degradation by the inclusion of 2′-fluoro modified pyrimidines [28], [29]. After selection, we sequenced 20 individual clones and found some that grouped into families based on sequence similarity. Several representative clones were screened for binding to GST-E7. As one of the highest affinity binders, aptamer A2 was selected for further study. The sequence and predicted secondary structures of A2 (lowest energy structures) are shown in Figure S1. Here, we show that this molecule inhibits cellular proliferation via induction of apoptosis in an HPV16-transformed cervical carcinoma cell line (SiHa) that actively expresses both E6 and E7 [30], but not in the control cell lines, HaCaT (a keratinocyte cell line) [31] and C33A (a cervical carcinoma cell line derived from an HPV-negative cancer) [32] or the HPV18 cell line, HeLa [33]. We also demonstrate that this aptamer binds to the region of E7 required for interaction with pRb and is capable of blocking the interaction of E7 with cellular pRb *in vitro*. Furthermore, transfection of the HPV16-positive cervical cancer cell line, CaSki [30], with this aptamer appeared to result in a loss of the E7 oncoprotein and in a rise in cellular pRb levels.

## Results

### Inhibition of Cellular Proliferation and Induction of Apoptosis in HPV16-Postitive Cells with Aptamer A2

**Figure 1:**
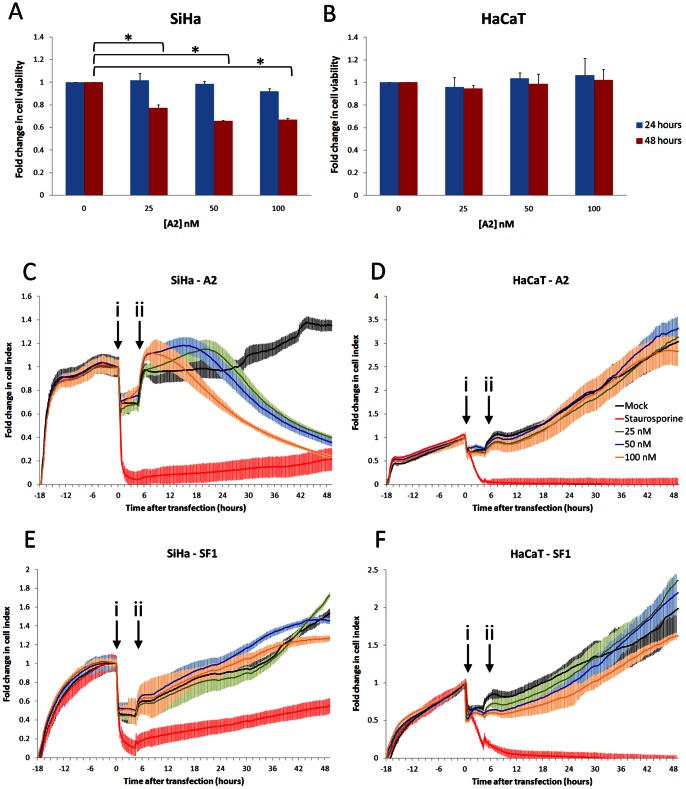
Reduction of cell growth in HPV16-transformed cells by aptamer A2. A2 was transfected into SiHa (A) or HaCaT (B) cells at concentrations of 0, 25, 50 or 100 nM. Cells were incubated for 24 or 48 hours and metabolically active cells were identified by MTT assay. Data are presented as fold change in cell growth compared with mock-treated cells. Standard errors of three separate experiments are shown. *indicates p<0.05. SiHa (C and E) or HaCaT (D and F) cells were seeded at 10,000 per well of a Roche E-plate and allowed to adhere for 18–21 hours. Cells were then transfected with 25, 50 or 100 nM of either A2 (panels C and D) or control aptamer SF1 (panels E and F) and incubated in serum-free media for 4 hours. Serum was added to a final concentration of 10% (v/v) and cells analysed by the Roche xCELLigence system for a further 48 hours. The average of 3 experiments and standard errors are shown. i = transfection with aptamer; ii = addition of serum.

We have previously reported the selection of RNA aptamers against the oncogenic HPV16 protein E7 and demonstrated that aptamer A2 binds to GST-E7 *in vitro* [14]. In order to assess A2-mediated effects on cells expressing E7, cell growth rates were analysed. SiHa cells were selected for this study as these cells constitutively express both E6 and E7. The cells are derived from a human cervical carcinoma and have 1 or 2 copies of the HPV16 genome integrated into chromosome 13 [30]. HaCaT (immortalised keratinocyte) cells were used as a negative control. Cells were transfected with up to 100 nM A2 and analysed after 24 or 48 hours by MTT assay. No significant changes in cell viability were measured after 24 hours, (Figure 1A), however, SiHa cells transfected with A2 had reduced cell viability after 48 hours, compared to mock-transfected cells (a reduction of between 22.9±3.0 and 34.4±0.9%). No alteration in growth was detected in HaCaT cells (Figure 1B).

The observations above were confirmed with the use of a real-time cell electronic sensing (RT-CES) system (xCELLigence, Roche), which measures cellular proliferation via the electrical impedance conferred on a microelectrode array [34], [35]. In brief, cells were allowed to adhere to microelectrode-coated 96-well plates for 18 hours prior to transfection with either A2 or an aptamer selected to an unrelated protein (the RNA-dependent-RNA polymerase of FMDV) as a negative control (referred to as SF1). This aptamer contains the same primer sequences as A2 and only differs in the N_30_ random region, thus sharing 61.5% similarity. The predicted lowest energy structures of both A2 and SF1 are shown in Figure S1. It should be noted that 2′F modifications can affect the pucker of the ribose which is not taken into account in this folding algorithm. Therefore, the predicted structures are an approximation and may not accurately represent aptamer structure in solution. Cellular proliferation rates were measured in triplicate as change in electrical impedance (cell index) over time (Figures 1C to F). Transfection was performed in serum-free media for 4 hours and corresponded with a rapid drop in impedance (arrowed, i). However, this returned to approximately the pre-transfection level, in most cases, upon addition of serum (arrowed, ii). Both SiHa and HaCaT cells mock-transfected with lipofection reagent alone continued to proliferate over time. However, the proliferation rate fell rapidly when cells were treated with staurosporine as an inducer of apoptosis [36], [37]. The subsequent small rise in cell index observed with SiHa cells is consistent with some cells surviving staurosporine treatment. Transfection of SiHa cells with aptamer A2 caused the cell index to decrease over time (Figure 1C). The effect was rapid in cells transfected with 100 nM A2, in which the decrease in cell index commenced at 8 hours post-transfection. After 24 hours the cell index had fallen by 32.6±4.4%. With reduced concentration of A2 there was delayed onset of effect; the cell index of SiHa cells transfected with 50 or 25 nM A2 did not begin to decrease until 18 and 22 hours post-transfection, respectively. This did not reach the levels seen with 100 nM A2. After 48 hours post-transfection, there was a dose-dependent decrease in cell index of 59.0±1.9%, 63.1±3.3% and 75.6±1.5% with 25, 50 and 100 nM A2, respectively. The slight increase in the cell index recorded immediately after recovery with serum is likely to reflect changes in cell morphology and adhesion consistent with the induction of apoptosis. Other studies using this system for the analysis of cytotoxic compounds have observed similar cell index profiles [38], [39], [40]. In contrast, HaCaT cells (which do not express E7) were unaffected at any concentration of A2 tested (Figure 1D). Furthermore, the negative control aptamer, SF1, had no significant effect on the proliferation of either SiHa or HaCaT cells (Figures 1E and F respectively).

**Figure 2:**
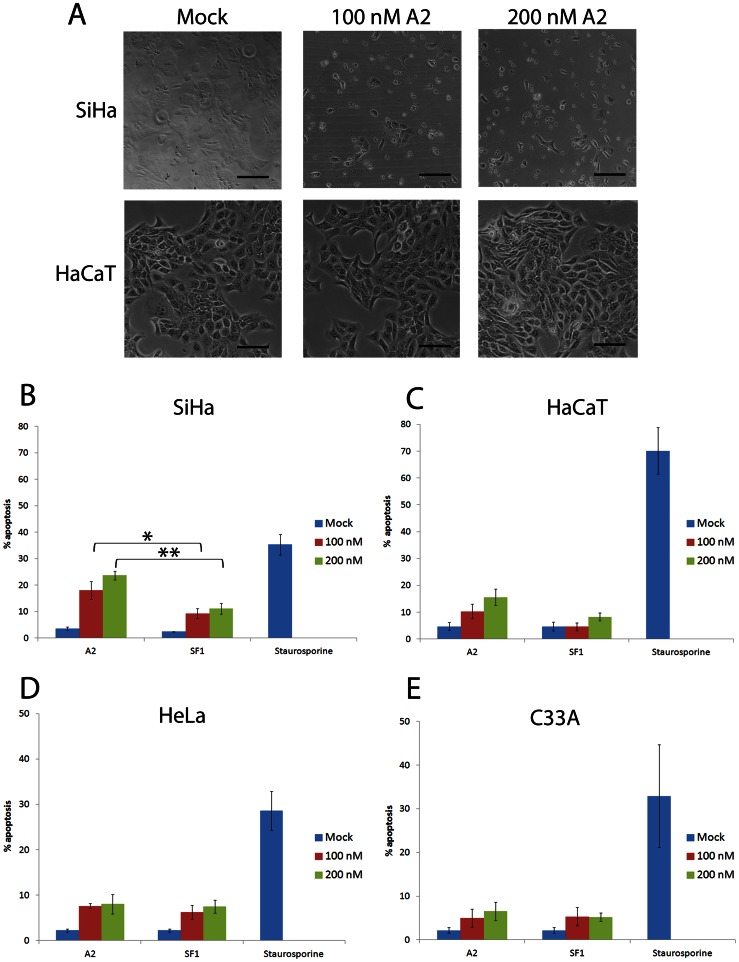
Cell death on transfection with aptamer A2. (A) SiHa or HaCaT cells were either mock transfected or transfected with 100 nM or 200 nM A2 and maintained for 48 hours. Shown are bright-field microscopy images. Scale bar: 100 µm. (B) SiHa, (C) HaCaT, (D) HeLa and (E) C33A cells were either mock-transfected, treated with staurosporine at 0.5 µM or transfected with either A2 or control aptamer SF1 to final concentration of 100 or 200 nM and analysed for apoptosis after 24 hours. Cells were dual-stained with FITC-conjugated annexin V and propidium iodide. Graphs show total % apoptosis and standard errors of at least three separate experiments. *indicates p<0.05, **indicates p<0.01.

The changes in cell index measured by RT-CES were greater than the changes in viability measured by MTT assay. This could reflect insensitivity of the latter, or that the real-time cell sensing assay may over-estimate reductions in cell growth because of changes in cell morphology. The appearance of SiHa cells after transfection with A2 was indicative of induction of apoptosis, whilst HaCaT cells appeared unaffected (Figure 2A). To quantify this observation, apoptosis assays were performed in which SiHa cells were transfected with increasing concentrations of aptamer (up to 200 nM). After 24 hours, samples were stained with FITC-conjugated annexin V and propidium iodide to identify apoptotic cells by flow cytometry. A small increase in apoptosis was induced in SiHa cells transfected with the control aptamer SF1, 9.25±1.9% and 11.2±2.0% at 100 nM and 200 nM, respectively (Figure 2B). However, the level of apoptosis in cells transfected with A2 was greater, with 18.0±3.4% and 23.7±1.7% apoptosis at 100 nM and 200 nM A2, respectively (Figure 2B). For both concentrations of A2 used, the level of total apoptosis increased in a dose-responsive manner and was significantly higher than that induced by SF1 (p<0.05 and p<0.01, respectively). This was due to an increase in both early and late apoptosis 24 hours after transfection with A2. The level of A2-induced apoptosis therefore accounts for a large proportion of the observed decrease in growth (33%) of SiHa cells at 24 hours post-transfection with 100 nM A2, shown in Figure 1C. Although there was a small increase in apoptosis seen in HaCaT cells, there was no significant difference in the levels induced by A2 or SF1 (Figure 2C), in contrast to that observed in SiHa cells. It should be noted that although HaCaT is an HPV-negative cell line, it is not derived from a cervical carcinoma. We therefore extended studies to include two additional cervical carcinoma cell lines: HeLa cells (HPV18-positive) and C33A cells (HPV-negative). Figures 2D and 2E demonstrate that both SF1 and A2 have a small effect on the level of apoptosis measured in both HeLa and C33A cells, however, neither of these increases was significantly different and are likely to have resulted from a non-specific response to the presence of the RNA. The data also indicates that there is HPV-type specificity of A2, appearing to having little effect on HPV18 E7-expressing cells, however further experiments would be necessary to confirm this. It should be noted that the innate immune response in HeLa cells has been shown to be much reduced compared to C33A cells [41]. Therefore, if the results seen here were due exclusively to innate immunity, we would have expected to see a greater effect in C33A than in HeLa cells. We would also have expected to see similar effects of SF1 and A2 in SiHa cells. In contrast, these data collectively suggest that the effect of A2 on SiHa cell proliferation is at least in part due to an induction of targeted apoptosis. As similar effects were seen in HaCaT, C33A and HeLa cells, HaCaT cells were used as a control cell line for further experiments.

### A2 Binds at the N-terminus of HPV16 E7

**Figure 3:**
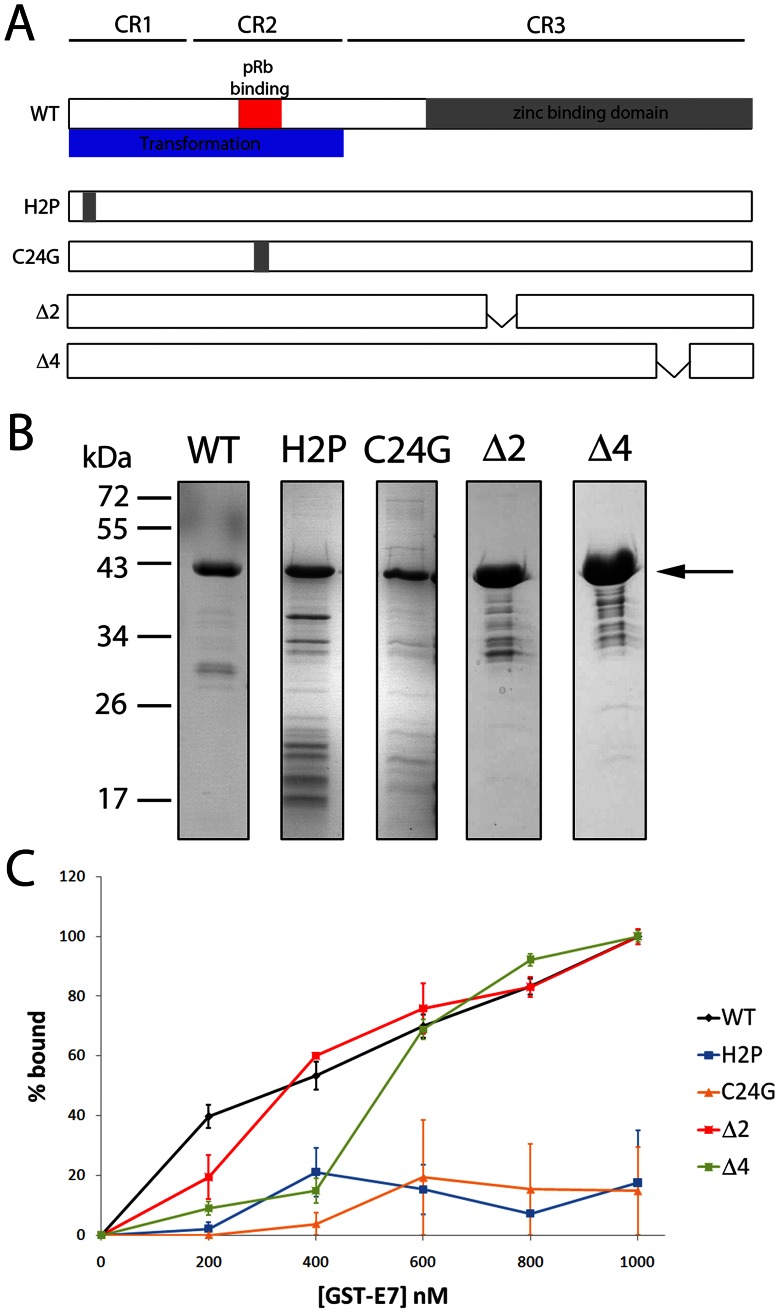
Binding of aptamer A2 to wild-type and mutant HPV16 E7 proteins. (A) Schematic representation of the HPV16 E7 protein indicating point mutations and deletions. (B) SDS-PAGE analysis of E7 proteins after expression, purification and binding to GM-beads. Samples were visualised by Coomassie staining. Arrow indicates full-length protein. (C) A2 was 5′ end-labelled with [γ^32^P]-ATP and incubated with increasing concentrations of bead-bound protein. GST-only beads were included to maintain a constant volume of beads. % bound RNA was quantified by scintillation counting. A2 was probed for binding to wild-type (WT) GST-E7, GST-E7-Δ2, GST-E7-Δ4, GST-E7 H2P and GST-E7 C24G. Data shown is the average of 3 replicates and standard error.

E7 has three conserved regions and has been shown to bind pRb via a LXCXE motif in conserved region 2 (CR2) (Figure 3A) [42], [43], [44]. In contrast, binding of E7 to p300 involves residue 2 in CR1 as well as residue 24 in the LXCXE motif [45] and the binding of TBP requires residues in the zinc-binding domain of CR3 [46]. To identify regions of the E7 protein involved in the interaction with A2, a panel of mutant GST-E7 proteins [45] was expressed in *E. coli*, purified (Figure 3B) and binding assays performed, as previously reported [14], [27], [47]. In brief, purified proteins were bound to glutathione-magnetic (GM) beads and incubated with [γ^32^P]-labelled A2. The percentage of bound aptamer was determined by scintillation counting of the bound and unbound fractions. Although maximal binding was not achieved in these experiments, by comparing the data in a qualitative manner it can be seen that binding of A2 to E7 was moderately affected by small deletions in the zinc-binding domain (Δ2 or Δ4), however, binding was abrogated by point mutations at residues 2 or within the LXCXE motif at position 24 (Figure 3C), indicating that A2 binds at the N-terminus of E7. Furthermore, the changes seen in A2-E7 binding as a result of point mutations/deletions in E7, provides support for the interaction between E7 and A2 being specific, rather than E7 acting as a general RNA-binding protein.

### Disruption of the Interaction between E7 and pRb by Aptamer A2 *in vitro*

**Figure 4:**
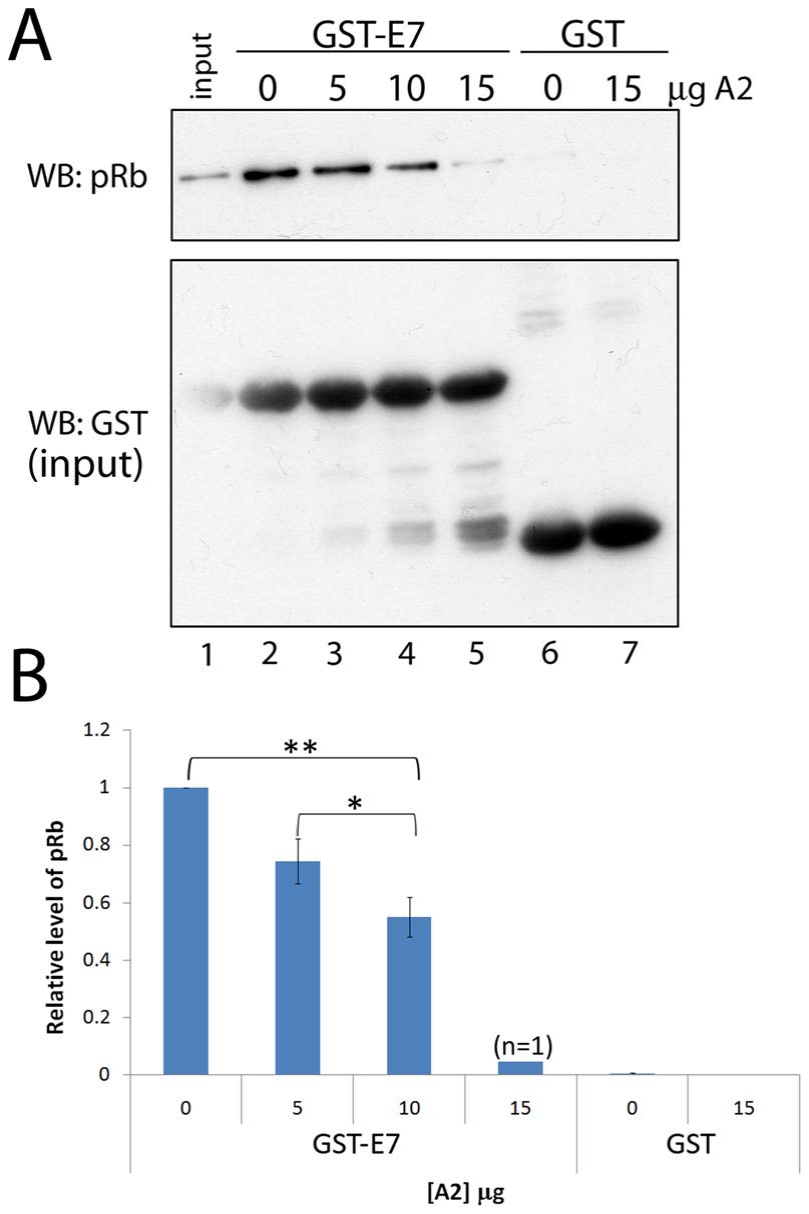
Disruption of the interaction between HPV16 E7 and pRb by aptamer A2 *in vitro*. (A) GST pull-down assay in the presence and absence of aptamer A2. 1 µg of GST-E7 (54 µM) bound to GM-beads was incubated with 300 µg of protein from a HaCaT cell lysate (as a source of pRb) for 1 hour at 4°C in the presence of 0, 5, 10 or 15 µg of A2. Bead-bound GST alone was incubated with either 0 or 15 µg (11 µM) A2. GST proteins and interacting proteins were isolated from the reaction and analysed by immunoblot using antibodies to pRb and GST. (B) Densitometric analysis of the interaction between GST-E7 and pRb in the absence and presence of A2, standard errors relating to three separate experiments are shown. *indicates p<0.05, **indicates p<0.01.

As both aptamer A2 and the pRb protein require residues in the N-terminal region of E7 for binding, we predicted that our aptamer might disrupt interactions between E7 and pRb. Indeed, our previous preliminary studies demonstrated that A2 had the ability to interfere with interactions between pRb and GST-E7 [14]. We therefore carried out further GST pull-down assays to quantify this effect (Figures 4A and B). For these experiments we used HaCaT cell lysate, as these cells do not express E7 and would therefore provide a source of pRb in the absence of endogenous E7 that could interfere with the binding assay. HaCaT lysate (lane 1) was incubated with GST-E7 (54 µM) immobilised onto GM-beads. In the absence of A2 (lane 2), pRb was precipitated by GST-E7, however, with increasing concentrations of A2 (lanes 3–5) the amount of pRb precipitated by GST-E7 was reduced. GST alone did not interact with pRb in either the absence or presence of A2 (lanes 6 and 7, respectively). These data confirm that A2 is able to disrupt the E7-pRb interaction *in vitro* in a dose-dependent manner, by up to 96% at the highest concentration tested (15 µg, 11 µM) (Figure 4B).

**Figure 5:**
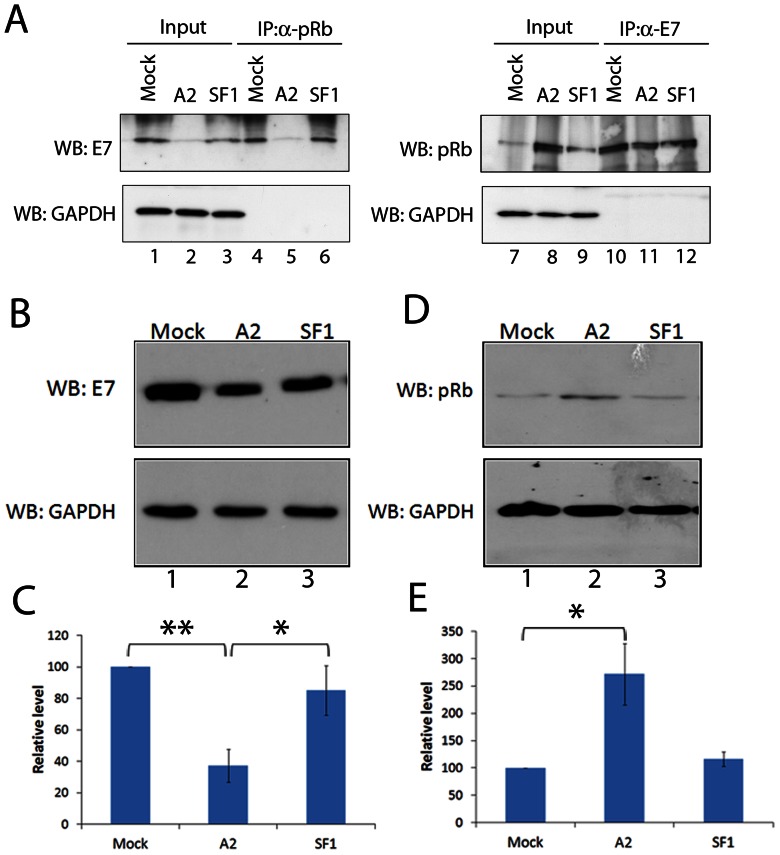
Reduction of HPV16 E7 protein levels in CaSki cells following transfection of aptamer A2. (A) Co-immunoprecipitation of pRb and HPV16 E7 in the presence or absence of aptamer. CaSki cells were either mock-transfected or transfected with A2 or SF1 at a final concentration of 100 nM and incubated for a further 24 hours before lysis and co-immunoprecipitation with 2 µg of either anti-pRb or anti-E7. Immunoblot analysis was performed for the detection of E7, pRb or GAPDH. Values of densitometric analysis and lane numbers are included below the gels for reference in the text. (B) CaSki cells were mock-transfected or transfected with either 100 nM A2 or SF1 (control aptamer) and incubated for 24 hours. Cells were lysed in Laemmli buffer. Immunoblot analysis was then performed to detect E7 and GAPDH. Lane numbers are included below the gels for reference in the text. (C) Graph shows the results and standard errors of 4 separate experiments, with E7 levels shown as % mock. *indicates p<0.05, **p<0.01. (D) CaSki cells were mock-transfected or transfected with either 100 nM A2 or SF1 (control aptamer) and incubated for 24 hours. Cells were lysed in Laemmli buffer. Immunoblot analysis was then performed to detect pRb and GAPDH. Lane numbers are included below the gels for reference in the text. (E) Graph shows the results and standard errors of 4 separate experiments, with pRb levels shown as % mock. *indicates p<0.05.

To further investigate the effect of A2 on the E7-pRb interaction, we performed co-immunoprecipitation assays in the presence or absence of aptamer. For this analysis, 100 nM A2 or SF1 was transfected into CaSki cells, a cervical cancer cell line containing several hundred copies of the HPV16 genome [30]. These cells express E7 at approximately a 6-fold higher level than SiHa cells (O. Watherston, pers. comm.). Of note, the high level of E7 expression in CaSki cells meant that it was not possible to deliver sufficient aptamer in order to detect statistically significant levels of apoptosis (Figure S2). However, the E7 level was sufficient for detection by immunoblotting and thus allowed the effect of aptamer to be determined. After transfection, cells were incubated for 24 hours, followed by preparation of cell lysates and immunoprecipitation with antibodies to E7 or pRb. The experiment therefore allows quantification of levels of E7 and pRb in two ways; by co-immunoprecipitation and by comparison of input levels after 24 hours incubation with aptamer. In both mock-transfected and SF1-transfected samples, efficient co-immunoprecipitation of E7 was observed using anti-pRb (Figure 5A, lanes 4 and 6, respectively). However, when 100 nM A2 was transfected into the cells, co-immunoprecipitation of E7 by anti-pRb was reduced by 80% (comparing lanes 4 and 5). This is mirrored by the reduction of the input level of E7 in cells transfected with A2 for 24 hours (Figure 5A, lanes 1–3). A2 transfection reduced the level of E7 in these cells by 75% (comparing lanes 1 and 2), whilst in cells which received the SF1 control, the level of E7 was reduced by only 18% (comparing lanes 1 and 3). The A2-mediated reduction in E7 levels correlated with an increase in cellular pRb, as the total level of pRb in cells which had been transfected with 100 nM A2 for 24 hours was increased 5-fold (comparing the pRb doublet in lanes 7 and 8). However, when the cell lysate was immunoprecipitated with anti-E7, there was no difference in the level of co-immunoprecipitation of pRb by anti-E7 in any of the treatments (lanes 10–12). This was possibly due to the high level of E7 expression in these cells, coupled with the concentration of E7 by antibody binding. To investigate this further, the level of E7 in CaSki cells in the presence or absence of aptamer was investigated. CaSki cells were transfected with aptamer, incubated for 24 hours and E7 levels then detected by immunoblot. Figure 5B shows that in cells that were either mock-transfected (lane 1) or transfected with the control aptamer SF1 (lane 3) the level of E7 was significantly higher than in cells transfected with A2 (lane 2). Densitometry analysis of 4 independent experiments (Figure 5C) showed that in CaSki cells transfected with A2, the level of E7 was reduced by 62.7±10.5% (lane 2), while in SF1-transfected cells the level was reduced by only 15.0±15.7% (lane 3). Together, these results indicate that during the 24 hour incubation with A2, E7 had been lost from the cells. Experiments were also performed to investigate the effects of A2 on levels of endogenous pRb. As a result of A2 transfection, pRb levels were elevated. Although there was a degree of variability in the level detected, on average, almost three-fold increase over mock transfection was measured (271.9±112.1%), as shown in Figures 5D and E, comparing lanes 1 and 2. Transfection with the control aptamer SF1 (lane 3) had little effect (116.2±26.5%).

## Discussion

RNA aptamers are currently employed as tools to investigate a variety of viral and cellular proteins [21] and also for development in diagnostics and as anti-viral agents [48], [49]. We have characterised an RNA aptamer (termed A2) selected against the HPV16 oncoprotein E7. Transfection of A2 into an HPV16-transformed cell line (SiHa) resulted in decreased cell viability by the induction of apoptosis. This aptamer was able to inhibit the interaction between HPV16 E7 and pRb *in vitro*. Upon further investigation, it appeared that transfection of CaSki cells with this molecule resulted in a reduction in E7 levels and that this loss appeared to correlate with an increase in the levels of pRb. However, it is not known whether A2 can affect the levels of other related pocket proteins such as p300.

Both pro- and anti-apoptotic effects of E7 have been reported in the literature. Our data are consistent with an anti-apoptotic effect of E7 in SiHa and CaSki cells. E7 is extremely unstable in the cell and is rapidly ubiquitinated and degraded via the proteasome [50], [51]. E7 proteins from high-risk HPVs have a high degree of intrinsic protein disorder (especially at the pRb-binding N-terminus) which is greater than that displayed by E7 proteins from low-risk types [52], [53], [54]. This flexibility may contribute to its ability to bind to a variety of proteins. It is possible that A2-mediated inhibition of the critical E7-pRb interaction could result in E7 instability and degradation, thus allowing restoration of pRb levels. Although it appears from the results reported here that A2 can result in disruption of the E7-pRb interaction *in vitro*, it is unclear if this also can occur in the context of the cell. It is therefore possible that A2 binding directly induces the loss of E7, resulting in a restoration of pRb levels. A previous study has shown that expression of constitutively-active pRb in cervical cancer cell lines resulted in the induction of apoptosis [55]. We suggest that the loss of E7 that was detected upon transfection of A2 resulted in a release of active pRb, ultimately resulting in an apoptotic response. It remains possible that the slight increases in apoptosis seen with the control aptamer SF1 are due to the presence of modified nucleotides or innate RNA sensing. However, these effects are small in comparison with the effects of A2 and therefore the main mode of action of A2 is unlikely to be a result of the innate immune response to aptamer RNA.

Interestingly, a similar result to that reported here has been observed with the use of an E7 siRNA. Although E7 protein levels were not measured directly, an increase in hypophosphorylated pRb was demonstrated which correlated with induction of apoptosis [56]. However, we (and others) have also observed different effects of E7 siRNAs e.g. growth arrest [57]. It should be noted that the mode of action of siRNA molecules is to interfere with protein synthesis at the translational level. As E6 and E7 are expressed from a single bicistronic mRNA, the use of siRNA can result in the down-regulation of both proteins and in off-target effects, possibly explaining the different results documented [58]. Although off-target effects remain a possibility here, we have demonstrated little effect of A2 in three HPV16-negative cell lines (HeLa, C33A and HaCaT). Using pull-down assays, we have also demonstrated that A2 had no effect on the interaction between p53 and E6 (data not shown/Belyaeva et al, in preparation).

Our data are also supported by several reports of the pro-apoptotic effects of E7-targeted peptides [59], [60], [61]. Furthermore, one of these molecules was demonstrated to act via reactivation of the pRb/E2F pathway, consistent with the effects of the RNA aptamer A2, described here. Although peptide aptamers can be costly to produce at high purity, together these studies demonstrate the power of these approaches in the development of potential novel therapeutics. Recently, a separate study has identified further RNA aptamers to HPV16 E7 [62]. Although these were not characterised in cells, this work further illustrates the potential of this technology. The use of E7 aptamers as specific molecular tools is therefore likely to be helpful in dissecting these pathways and in understanding the complex roles of E7.

Our previous studies have demonstrated that small sequence variations in A2 have affected the ability of the aptamer to affect the E7-pRb interaction [14]. Further characterisation of A2 may identify a minimal binding region and thus allow generation of a smaller molecule, cheaper and easier to produce. Work is ongoing to characterise other E7 aptamers from the selected pool [14] and aptamers to HPV16 E6, as targeting both oncoproteins is likely to have an additive effect. Studies are also on-going to further investigate the pathway of E7 loss, the specificity of the aptamers and aptamer delivery into primary cells. Aptamer technology thus has the potential to provide new insights into the multiple molecular interactions performed by these small viral oncoproteins.

## Materials and Methods

### Protein Expression and Purification

Expression and purification of GST-E7 was undertaken following our previous published protocols [14]. Expression vectors for E7 with point mutations or deletions were also described previously [46], [63].

### Cell Culture and Antibodies

SiHa [64], CaSki [65], HeLa (HPV-positive human cervical cancer-derived) [66], C33A (HPV-negative human cervical cancer-derived) [67] and HaCaT (immortalised keratinocyte) [31] cells were maintained in DMEM supplemented with 10% (v/v) FCS, 100 units/ml penicillin, 0.1 mg/ml streptomycin and 1% (w/v) glutamine at 37°C and 5% (v/v) CO_2_. Antibodies used were anti-pRb (4H1, New England Biolabs and 1F8, AbCam), anti-16E7 (NM2, Santa Cruz), anti-GST (P1A12, Cambridge Bioscience), rabbit anti-pRb (Ab-2, Merck Chemicals) and anti-GAPDH (Sigma Aldrich).

### Aptamer Synthesis

*In vitro* selections were performed using the Biomek 2000 Automated Workstation (Beckman Coulter) as described previously [14]. *In vitro* transcription reactions were carried out including 2′-fluoro-UTP and 2′-fluoro-CTP (TriLink Biotechnologies), using a mutant T7 RNA polymerase [68], according to the method reported previously [14]. A2 and SF1 share 5′ and 3′ regions i.e. GGGAAUGGAUCCACAUCUACGAAU–N_30_–UUCACUGCAGACUUGACGAAGCUU. Sequences of the N_30_ regions were CCCUUCAUCAUUAACCCGUCCACGCGC and UCGGCUCAAAAAUACGUCCGCACCAUACA for A2 and SF1, respectively.

### Cell Proliferation Assays

- Measurement of cell viability was undertaken using the MTT assay. Cells were seeded at 10^4^ per well of a 96-well plate and incubated for 24 hours prior to transfection with increasing concentrations of aptamer (0, 25, 50 and 100 nM). Cells were maintained for a further 48 hours at 37°C, followed by incubation with 20 µl of 5 mg/ml 3-(4,5-Dimethylthiazol-2-yl)-2,5-diphenyltetrazolium bromide (MTT) in PBS for 4 hours at 37°C. Formazan crystals were dissolved by incubation with acidic isopropanol (96% isopropanol/4% 1M HCl) for one hour at 37°C. Absorbance was measured at λ = 570 nm with background subtraction at 630 nm.
- Real-time monitoring of cellular proliferation was performed using the xCELLigence system (Roche, UK). Cells were seeded at 10^4^ per well of a micro-electrode coated 96-well plate and allowed to adhere for 17–21 hours. Cells were either mock-transfected or transfected with aptamer using Oligofectamine (Invitrogen) and maintained at 37°C with monitoring every 15 minutes. Automatic analysis by the RTCA software generated a measurement of cell proliferation based on the electrical impedance conferred on the micro-electrodes by the presence of cells (termed cell index).

### Apoptosis Assays

Cells (1.5×10^5^) were seeded into 4 cm^2^ wells 24 hours prior to transfection with up to 200 nM of A2 or SF1 as described above and maintained at 37°C for 24 hours. Cells were harvested by trypsinisation, washed twice with PBS and suspended in ice cold annexin V buffer (10 mM HEPES-KOH (pH 7.4), 140 mM NaCl and 2.5 mM CaCl_2_) with FITC-conjugated annexin V and incubated on ice for 15 minutes. Cells were co-stained with propidium iodide at 50 µg/ml in PBS and analysed using the FACSCalibur and Cellquest Pro software (Becton Dickinson).

### Aptamer Binding Assays

Radiolabelled aptamer RNA was generated by 5′ end-labelling with [γ^32^P]-ATP, unincorporated nucleotides were removed by column purification (NucAway spin column, Ambion). Labelled RNA was incubated with protein-loaded GM-beads at a final concentration of 1 nM for 30 minutes at room temperature. Protein-loaded beads and any bound RNA were isolated from the reaction mixture and the supernatant transferred to scintillation fluid (unbound fraction). Beads were washed three times with PBS and suspended into scintillation fluid (bound fraction). The method is based on our previously reported protocols [14], [27], [47].

### GST Pull-down Assays

GST and GST-E7 proteins were bound to GM-beads and cell lysates prepared as described previously [14]. After incubation for 1 hour at 4°C, beads were isolated, washed and samples analysed by immunoblotting using anti-GST and mouse anti-pRb.

### Co-immunoprecipitation Assays

Cells (3×10^5^) in 6-well plates were transfected with 100 nM aptamer using Oligofectamine. Cells were lysed in RIPA buffer and cleared by centrifugation. Lysate was incubated with 2 µg of anti-E7 or anti-pRb overnight at 4°C with shaking. Antibody-lysate mixtures were centrifuged to remove precipitates followed by incubation with 20 µl of a 50% (v/v) protein G sepharose slurry (Sigma) for 2 hours. Sepharose was collected by centrifugation and washed with RIPA buffer three times before re-suspension in SDS-PAGE loading buffer and analysis by immunoblotting.

### Statistical Analysis

Standard errors are included, and a student’s T-test was performed to obtain p-values, where appropriate.

## Supporting Information

Figure S1 **Structure predictions for aptamers A2 and SF1 as calculated by Mfold** [**69**] **.** Three structures were predicted for A2, with 2 for SF1. ΔG values, as calculated by Mfold, are given.
(TIF)

Figure S2 **Apoptotic response of CaSki cells to aptamer.** CaSki cells were either mock-transfected, treated with staurosporine at 0.5 µM or transfected with either A2 or control aptamer (DB) to final concentration of 200 nM and analysed for apoptosis after 24 hours. Cells were dual-stained with FITC-conjugated annexin V and propidium iodide. Graphs show total % apoptosis. Standard error bars are shown were appropriate.
(TIF)
