# Supplementary figures and images for: An RNA Aptamer Provides a Novel Approach for the Induction of Apoptosis by Targeting the HPV16 E7 Oncoprotein
Source: PLoS One. 2013 May 30;8(5):e64781. doi: 10.1371/journal.pone.0064781 (PMC3667794; doi:10.1371/journal.pone.0064781)

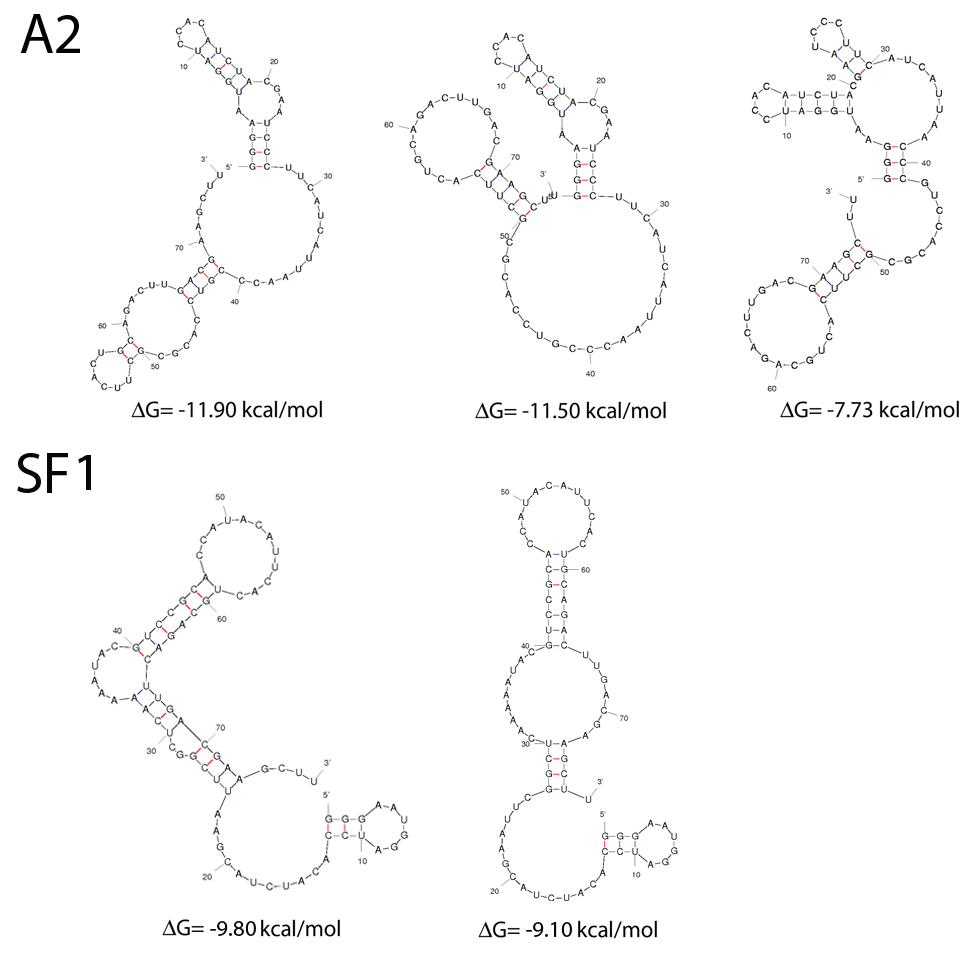

Supplement: Figure S1 — Structure predictions for aptamers A2 and SF1 as calculated by Mfold [69] . Three structures were predicted for A2, with 2 for SF1. ΔG values, as calculated by Mfold, are given. (TIF) [file pone.0064781.s001.tif]

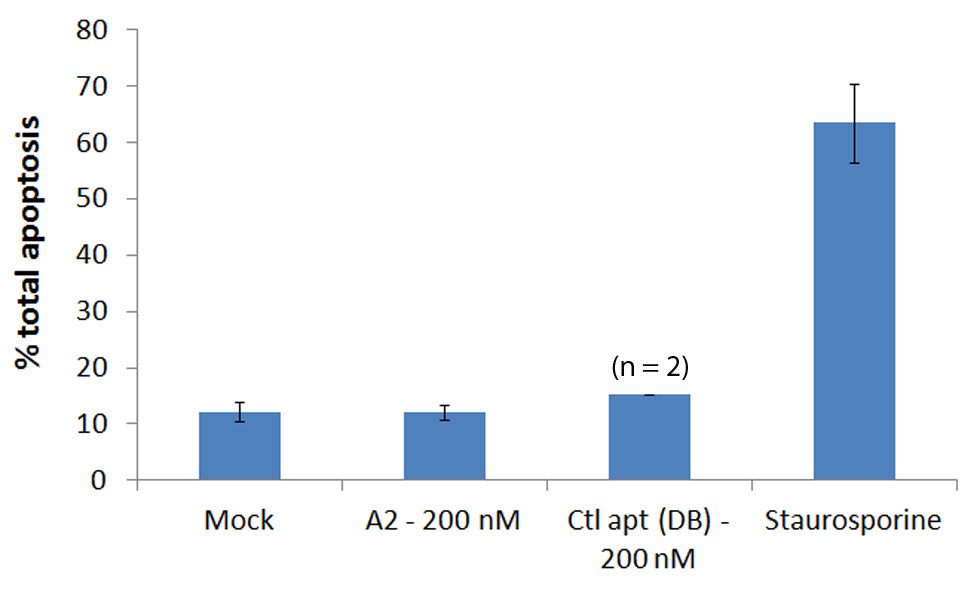

Supplement: Figure S2 — Apoptotic response of CaSki cells to aptamer. CaSki cells were either mock-transfected, treated with staurosporine at 0.5 µM or transfected with either A2 or control aptamer (DB) to final concentration of 200 nM and analysed for apoptosis after 24 hours. Cells were dual-stained with FITC-conjugated annexin V and propidium iodide. Graphs show total % apoptosis. Standard error bars are shown were appropriate. (TIF) [file pone.0064781.s002.tif]
